# HDAC Inhibition Improves the Sarcoendoplasmic Reticulum Ca^2+^-ATPase Activity in Cardiac Myocytes

**DOI:** 10.3390/ijms19020419

**Published:** 2018-01-31

**Authors:** Viviana Meraviglia, Leonardo Bocchi, Roberta Sacchetto, Maria Cristina Florio, Benedetta M. Motta, Corrado Corti, Christian X. Weichenberger, Monia Savi, Yuri D’Elia, Marcelo D. Rosato-Siri, Silvia Suffredini, Chiara Piubelli, Giulio Pompilio, Peter P. Pramstaller, Francisco S. Domingues, Donatella Stilli, Alessandra Rossini

**Affiliations:** 1 Institute for Biomedicine, Eurac Research, 39100 Bolzano, Italy (affiliated institute of the University of Lübeck, 23562 Lübeck, Germany); viviana.meraviglia@eurac.edu (V.M.); cristiflorio@gmail.com (M.C.F.); benedetta.motta@eurac.edu (B.M.M.); corrado.corti@eurac.edu (C.C.); christian.weichenberger@eurac.edu (C.X.W.); yuri.delia@eurac.edu (Y.D.); Marcelo.RosatoSiri@eurac.edu (M.D.R.-S.); silvia.suffredini@alice.it (S.S.); chiara.piubelli@sacrocuore.it (C.P.); peter.pramstaller@eurac.edu (P.P.P.); francisco.domingues@eurac.edu (F.S.D.); 2 Department of Chemistry, Life Sciences and Environmental Sustainability, University of Parma, 43124 Parma, Italy; leonardo.bocchi@unipr.it (L.B.); monia.savi@unipr.it (M.S.); 3 Department of Comparative Biomedicine and Food Science, University of Padova, 35020 Legnaro (Padova), Italy; roberta.sacchetto@unipd.it; 4 Vascular Biology and Regenerative Medicine Unit, Centro Cardiologico Monzino, IRCCS, 20138 Milano, Italy; giulio.pompilio@ccfm.it; 5 Dipartimento di Scienze Cliniche e di Comunità, Università degli Studi di Milano, 20122 Milano, Italy

**Keywords:** SERCA2, acetylation, HDAC inhibition, ATPase activity, calcium transients, cardiomyocyte mechanics

## Abstract

SERCA2a is the Ca^2+^ ATPase playing the major contribution in cardiomyocyte (CM) calcium removal. Its activity can be regulated by both modulatory proteins and several post-translational modifications. The aim of the present work was to investigate whether the function of SERCA2 can be modulated by treating CMs with the histone deacetylase (HDAC) inhibitor suberanilohydroxamic acid (SAHA). The incubation with SAHA (2.5 µM, 90 min) of CMs isolated from rat adult hearts resulted in an increase of SERCA2 acetylation level and improved ATPase activity. This was associated with a significant improvement of calcium transient recovery time and cell contractility. Previous reports have identified K464 as an acetylation site in human SERCA2. Mutants were generated where K464 was substituted with glutamine (Q) or arginine (R), mimicking constitutive acetylation or deacetylation, respectively. The K464Q mutation ameliorated ATPase activity and calcium transient recovery time, thus indicating that constitutive K464 acetylation has a positive impact on human SERCA2a (hSERCA2a) function. In conclusion, SAHA induced deacetylation inhibition had a positive impact on CM calcium handling, that, at least in part, was due to improved SERCA2 activity. This observation can provide the basis for the development of novel pharmacological approaches to ameliorate SERCA2 efficiency.

## 1. Introduction

The Sarco(Endo)plasmic Reticulum (SR) Ca^2+^ ATPase (SERCA) is a Ca^2+^ pump that uses the energy of ATP hydrolysis to translocate two Ca^2+^ ions from the cytosol to the SR lumen. It is composed of a single polypeptide weighing approximately 110 kDa and organized into four different domains: one transmembrane domain composed of ten alpha-helices, one nucleotide (ATP) binding site, one phosphorylation/catalytic domain that drives ATP hydrolysis, and one actuator domain involved in the gating mechanism that regulates calcium binding and release [1,2]. In vertebrates, three different SERCA genes have been identified so far, showing high degree of conservation among species: *ATP2A1*, *ATP2A2* and *ATP2A3*, encoding respectively for the SERCA1, SERCA2 and SERCA3 proteins. Thanks to alternative splicing mechanisms, they can give rise to different protein isoforms, whose expression is regulated during development and in a tissue specific manner [3]. SERCA2a is the most expressed isoform in cardiac muscle [3] and is the responsible for the level of SR Ca^2+^ load and the majority of the cytosolic calcium removal after contraction (70% in human, up to 90% in mouse and rats), thus representing a key player for cardiomyocyte (CM) relaxation [4]. Alterations in SERCA2a are recognized among the major factors contributing to ventricular dysfunction in several heart diseases, like diabetic cardiomyopathy [5,6] and heart failure [7,8].

Owing its essential role in cardiac physiology, the mechanisms regulating SERCA2a have been extensively studied [9]. Evidence has been provided that the pump activity can be directly modulated by several post-translational modifications [10]. Specifically, sumoylation [11] and glutathionylation [12] increase the activity, while glycosylation [13] and nitration [14] seem to decrease SERCA2a function. A recent study by Foster and coworkers [15] identified three acetylation sites within the structure of SERCA2a. However, the potential impact of SERCA2a acetylation on intracellular Ca^2+^ cycling has never been evaluated. 

The enzymes responsible for protein acetylation and deacetylation are histone acetyltransferases (HATs) and histone deacetylases (HDACs), respectively. HATs and HDACs act not only on histones, but also on other nuclear and cytoplasmic proteins, such as transcription factors and structural proteins [16].

The acetylation balance carried out by HATs and HDACs plays a role in the physiology of adult CMs. For instance, it has been demonstrated that HDAC inhibition prevented ventricular arrhythmias and restored conduction defects of the heart in a model of dystrophic mice [17].

The specific aim of the present work was to investigate the impact of deacetylation inhibition on SERCA2 activity. Specifically, adult ventricular CMs isolated from normal rat hearts were exposed to the HDAC general inhibitor suberanilohydroxamic acid (SAHA, or Vorinostat). Additionally, three stable lines of HEK cells were produced to express the human SERCA2a either wild type or with mutations mimicking constitutive acetylation/deacetylation on a selected lysine residue.

In this manuscript, we demonstrate for the first time that HDAC inhibition promotes improved efficiency in cytosolic Ca^2+^ removal, along with increased SERCA2 acetylation and ATPase activity.

## 2. Results

### 2.1. Effect of SAHA Treatment on SERCA2a Acetylation and Function in Control Rat Cardiomyocytes 

**Figure 1:**
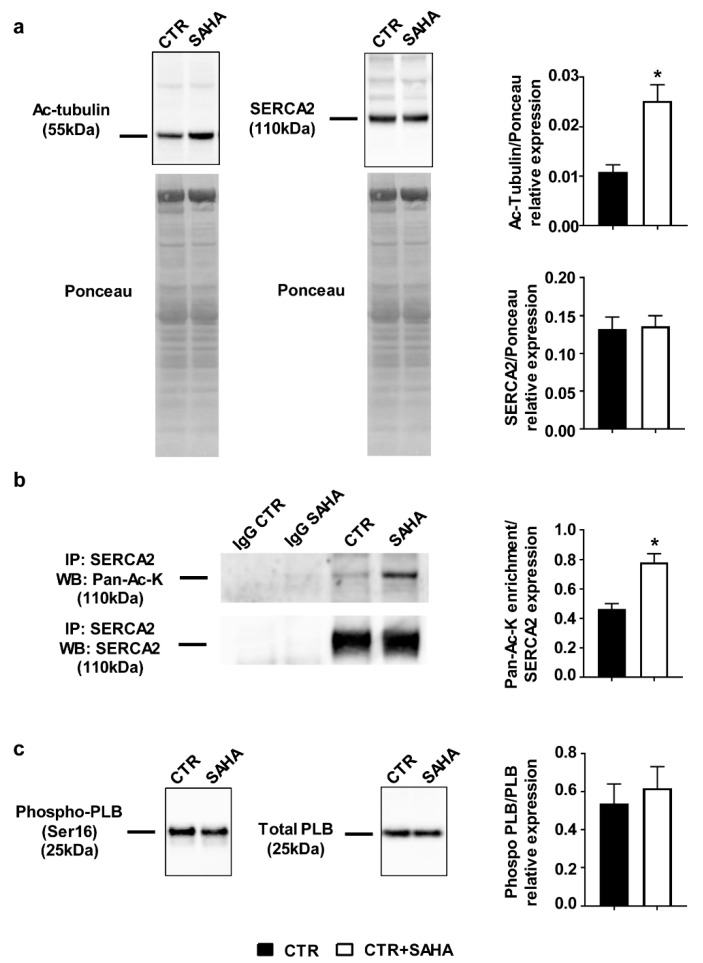
Effect of SAHA treatment on cardiomyocytes isolated from adult rat hearts. (**a**) Western blot panels (**on the left**) and densitometric analysis (**on the right**) showing SERCA2 and Ac-Tubulin protein expression after SAHA treatment (*n* = 7). Mann–Whitney *U*-test: * *p* < 0.005 vs. CTR; (**b**) Immunoprecipitation experiments and densitometric analysis (**on the right**) indicating higher acetylation level of SERCA2 after SAHA treatment (*n* = 5). Mann–Whitney *U*-test: * *p* < 0.005 vs. CTR; (**c**) Western blot analysis showing the expression of phosphorylated phospholamban (Phospho-PLB, Ser16) compared to total phospholamban (PLB) in adult rat CMs after SAHA treatment (*n* = 7).

Cardiomyocytes (CMs) isolated from normal adult rat hearts were used as model to investigate whether SAHA-promoted HDAC inhibition can impact SERCA2a acetylation and function. Following enzymatic dissociation, CMs were either untreated (CTR) or incubated with 2.5 µM SAHA (CTR+SAHA) for 90 min. As expected [18], SAHA administration induced higher tubulin acetylation level (Figure 1a).

Co-immunoprecipitation experiments revealed that SAHA increased SERCA2 acetylation level (Figure 1b and Figure S1), without inducing significant changes in SERCA2 protein expression (Figure 1a). The ratio of phosphorylated phospholamban (PLB)/total phospholamban (Figure 1c) remained unchanged.

**Figure 2:**
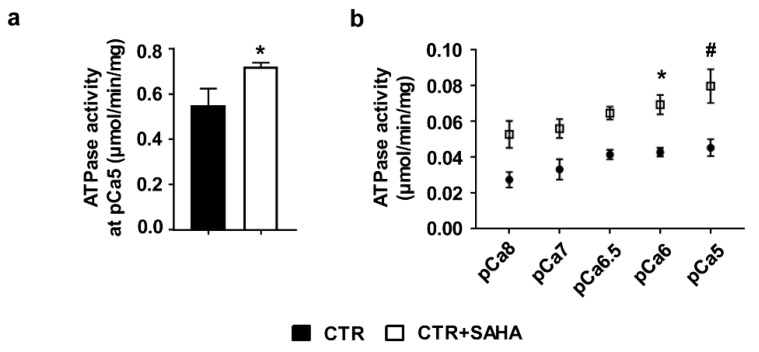
Effect of SAHA treatment on SERCA2 ATPase activity evaluated in cardiomyocytes isolated from adult rat hearts and HL-1 cells. (**a**) ATPase activity assay performed on microsomes isolated from adult rat CMs either untreated or treated with SAHA at pCa5. Experiments were performed on 3 independent CM sets per group and repeated twice. Unpaired Student’s *t*-test: * *p* < 0.005 vs. CTR; (**b**) ATPase activity assay performed on microsomes isolated from HL-1 cells at different pCa. Each point represents the mean ± SEM of at least 4 independent experiments; Two-way ANOVA followed by Sidak’s multiple comparison: * *p* < 0.05 vs. SAHA pCa6; # *p* < 0.05 vs. SAHA pCa5. All data are presented as mean ± SEM.

To investigate whether increased acetylation could also affect SERCA2 functional properties, ATPase activity was measured on microsomes [19] isolated from both CTR and CTR+SAHA CMs. HDAC inhibition resulted in an increase of ATPase activity when microsomes were exposed to 10 µM calcium concentration (corresponding to pCa5) [20,21] (Figure 2a).

In order to confirm our result on SERCA2 functional properties, we decided to perform an additional set of experiments measuring ATPase activity on microsomes isolated from HL-1 cells, derived from the AT-1 mouse atrial cardiomyocyte tumor lineage. These cells partially maintain an adult cardiac phenotype and are able to contract [22]. The calcium-dependence of ATPase activity on HL-1 cells, either untreated (CTR) or treated for 90 min with 2.5 µM SAHA, was analyzed at different calcium concentration (from pCa8 to pCa5). ATPase activity of microsomes extracted from HL-1 cells was increased after SAHA treatment in comparison with CTR and the difference reached statistical significance when microsomes were exposed to 1 and 10 µM calcium concentration (corresponding to pCa6 and pCa5, respectively; Figure 2b).

### 2.2. Effect of SAHA Treatment on Calcium Transients and Cell Mechanics in CMs Isolated from Adult Rat Hearts

**Figure 3:**
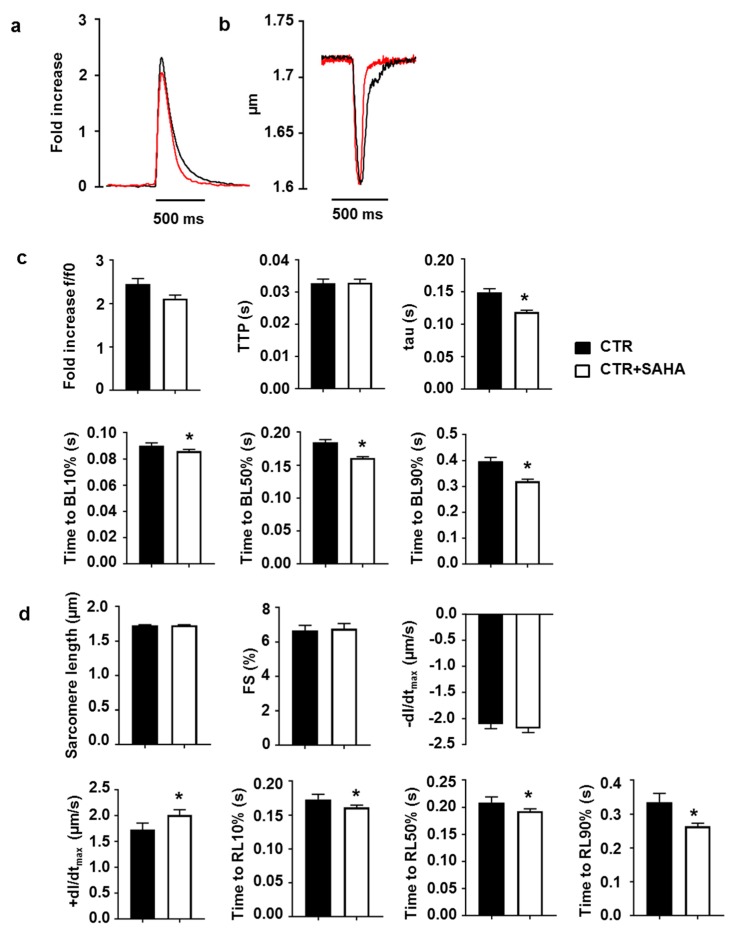
Effect of SAHA treatment on calcium transients and cell mechanics in CMs isolated from adult rat hearts. Representative examples of calcium transients (**a**) normalized traces: fold increase and sarcomere shortening (**b**) recorded from CTR (black line) and CTR+SAHA (red line) ventricular myocytes. (**c**) Calcium transient parameters: amplitude of calcium transient (expressed as calcium peak fluorescence normalized to baseline fluorescence, f/f0: fold increase), time to peak of the calcium transient (TTP), time constant of the rate of intracellular Ca^2+^ clearing (tau), and recovery phase of Ca^2+^ transients indicated as time to 10% (BL10), 50% (BL50) and 90% (BL90) of fluorescence signal decay. (**d**) Cell mechanics: diastolic sarcomere length and fraction of shortening (FS), maximal rates of shortening (−dl/dt_max_) and re-lengthening (+dl/dt_max_), time of re-lengthening at 10% (RL10), 50% (RL50) and 90% (RL90). All data are presented as mean ± SEM and analysed using General Linear Model (GLM) ANOVA for repeated measurements (*n* = 45 untreated CMs, *n* = 70 CMs treated with SAHA): * *p* < 0.005 vs. CTR.

We then investigated whether SAHA treatment affected CM functional parameters that directly depend on SERCA2 activity, namely calcium transients and cell contractility. The amplitude and the time to peak (TTP) of the calcium transient were comparable in CTR and CTR+SAHA groups, while the rate of cytosolic calcium clearing was significantly higher in SAHA-treated cardiomyocytes (Figure 3a,c). Specifically, SAHA induced a 21% decrease in the time constant tau, as well as a significant reduction in the time to 10%, 50% and 90% of fluorescence signal decay (BL10, BL50, BL90; Figure 3c). Consistent with this finding, SAHA also affected CM mechanics during the re-lengthening phase, as documented by the significant increase in the maximal rate of re-lengthening (+dl/dt_max_, approximately 16%) associated with a decrease in the time to 10%, 50% and 90% of re-lengthening (Figure 3b,d). Conversely, the average diastolic sarcomere length, the fraction of shortening and the maximal rate of shortening were comparable in CTR and CTR+SAHA cardiomyocytes (Figure 3d). Of note, SAHA exposure, at the conditions used in the present manuscript (90 min, 2.5 µM), did not affect cardiomyocytes diastolic nor systolic calcium concentration assessed by Fura-2 dye (Figure S2).

### 2.3. Nε-Lysine Acetylation Sites of Human SERCA2a

**Figure 4:**
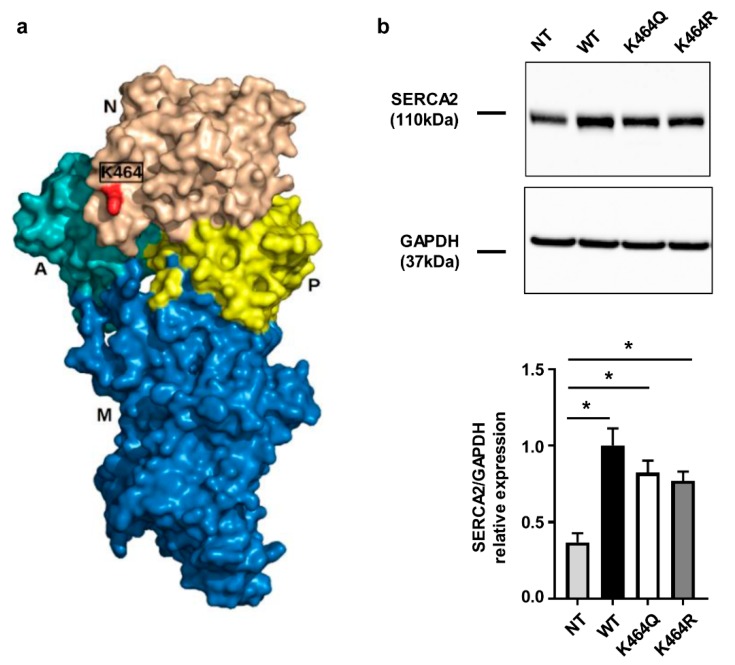
Analysis of human SERCA2a mutants transfected in HEK cells: bioinformatics prediction and protein expression. (**a**) Molecular surface view of predicted hSERCA2a. Domains are visualized in different colors: Actuator (A) in light blue, transmembrane (M) in dark blue, phosphorylation (P) in yellow and nucleotide-binding (N) in beige. K464 is highlighted in red; (**b**) Western blot analysis of SERCA2 expression in stable transfected HEK cells. Densitometry is reported in the bar graph (*n* = 8). Ordinary one-way ANOVA followed by Bonferroni’s multiple comparison: *p* < 0.005 vs. NT.

To support the hypothesis that direct acetylation can boost SERCA2a function, we searched in PHOSIDA and PhosphoSitePlus for reported Nε-lysine acetylation sites in human SERCA2. PHOSIDA Post Translation Modification Database reports acetylation at K464, while PhosphoSitePlus reports acetylation at K31, K33, K218, K464, K476 and K514. All these residues are mostly conserved in mammals (Figure S3). Out of these candidates, site K464 was selected as the target for further analysis given two previous independent reports identifying K464 as an acetylation site in human SERCA2 [23,24]. Homology modeling results indicate that K464 is solvent accessible at the surface of the nucleotide binding-domain (Figure 4a and Figure S4).

To assess the impact of Nε-lysine acetylation on hSERCA2a function, two different mutants were generated where lysine in position 464 was changed into glutamine (Q) or arginine (R) to mimic constitutive acetylation or constitutive deacetylation, respectively [15]. The expression of either the hSERCA2a wild type form (WT) and the mutant forms (K464Q/R) was induced in HEK human cells (Figure 4b), expressing lower levels of the SERCA2 isoform compared to other commonly used human cell lines, such as HeLa. SERCA2 expression was significantly increased in all stable transfected HEK cells compared to non-transfected cells (NT; Figure 4b). However, although the observed difference did not reach statistical significance, the expression of SERCA2 appeared lower in both mutants compared to the HEK transfected with WT (Figure 4b).

### 2.4. Effects of K464 Mutation on hSERCA2a ATPase Activity and HEK Calcium Transients

**Figure 5:**
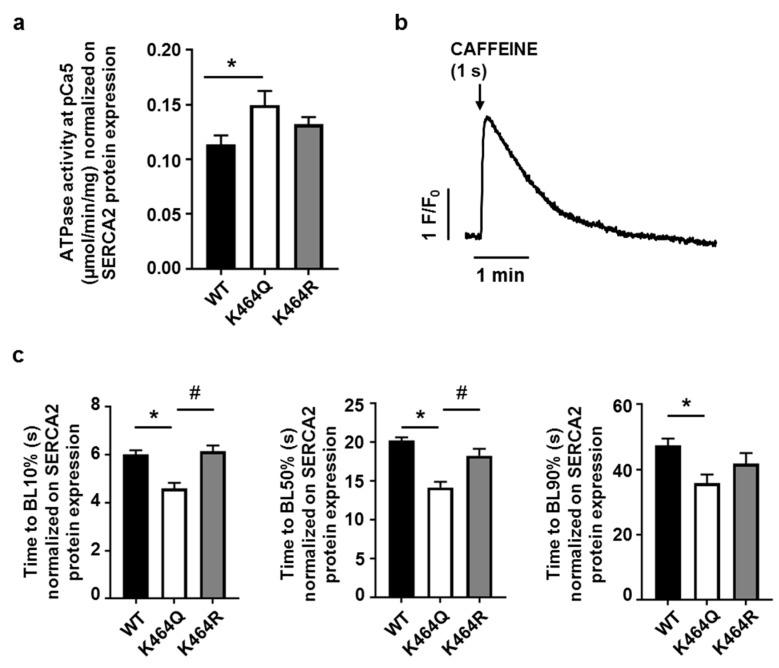
ATPase activity on microsomes and calcium transients evaluated in HEK cells transfected with human SERCA2a mutants. (**a**) ATPase activity assay performed on microsomes isolated from stable transfected HEK cells at pCa5 normalized on SERCA2 protein expression. Experiments were performed on 4 independent HEK sets per group and repeated twice. Ordinary one-way ANOVA followed by Bonferroni’s multiple comparison: *p* < 0.005 WT vs. K464Q. All data are presented as mean ± SEM; (**b**) Representative trace of the rise in intracellular calcium concentration evoked by a caffeine pulse in untransfected HEK cells; (**c**) Calcium transients in SERCA2a K464 mutants normalized on SERCA2 protein expression. K464 is mutated either into Q or R, mimicking constitutive acetylation and deacetylation, respectively (*n* = 99 WT, *n* = 69 K464Q and *n* = 36 K464R). Time to 10% (BL10) and time to 90% (BL90) were analysed by Kruskal-Wallis followed by Dunn’s multiple comparisons test: * *p* < 0.005 WT vs. K464Q, # *p* < 0.005 K464Q vs. K464R. Time to 50% (BL50) was analysed by ordinary one-way ANOVA followed by Bonferroni’s multiple comparisons test: * *p* < 0.005 WT vs. K464Q, # *p* < 0.005 K464Q vs. K464R. All data are presented as mean ± SEM.

The effects of mutagenesis were evaluated on ATPase activity measured in the microsomal fraction isolated from transfected HEK cells. In order to take into consideration the possible impact of different level of protein expression on hSERCA2a function, the values of ATPase activity recorded for each sample were normalized to the correspondent SERCA2 expression evaluated by Western blot on cells obtained from the same sample (Figure 4b). Taking into account the variable amount of SERCA2 expression in each cell line and for each experiment, we observed that SERCA2 function was significantly ameliorated in mutants where K464 were mutated into Q compared with WT (Figure 5a). Conversely, the K464R mutant did not show any differences in comparison with the WT (Figure 5a). Thus, constitutive acetylation of hSERCA2a K464 improved hSERCA2a ATPase activity, while K464 mutation into R (constitutive deacetylation) did not have a significant effect (Figure 5a). Importantly, untransfected HEK cells responded with a transient increase of intracellular calcium concentration to caffeine puff (1 s, 10 mM, Figure 5b). Therefore, we further evaluated the effects of SERCA2a mutagenesis on calcium transients induced by caffeine in HEK cells loaded with the calcium sensitive dye Fluo-4. Again, in order to consider the effects of different SERCA2 protein levels in HEK mutants, calcium transients were corrected on the mean SERCA2 expression detected by Western Blot in the correspondent cell line. The constitutive acetylation of SERCA2 K646Q promoted an acceleration of Ca^2+^ transient recovery phase at BL10%, BL50% compared to WT and K464R (Figure 5c), thus indicating that constitutive acetylation of K464 has a positive impact on hSERCA2a function.

## 3. Discussion

SERCA2 activity has been recently suggested to be affected by acetylation/deacetylation mechanisms [11,15], although no experimental evidence has so far been provided. In the present work, we show that HDAC inhibition promotes SERCA2 acetylation. This is associated with a significant increase of microsomal ATPase activity (which is recognized to be mainly due to SERCA2 function [25]) and a significant amelioration of calcium clearing parameters measured in CMs isolated from normal adult rats. Our results indicate for the first time a relation between HDACs, SERCA2 acetylation and its function. In addition, lysine 464 has been mutated in the human SERCA2a (hSERCA2a) coding sequence transfected in a HEK stable expression system, in order to provide evidence for a causal association between SERCA2 deacetylation and the ATPase activity of the pump.

In our CM experimental model, SERCA2 acetylation was promoted by SAHA (Vorinostat), a general HDAC inhibitor, acting on class I and II HDACs [26]. SAHA use was approved by the US FDA in October 2006 for the treatment of refractory cutaneous T-cell lymphoma [27]. Although several in vitro studies [28,29] have reported that 2.5 µM SAHA is able to inhibit the growth of cancer cells, it has also been reported that 2 µM SAHA is able to reduce CM death after simulated ischaemia/reperfusion injury [30]. In our work, the decision to treat adult CMs with 2.5 µM SAHA for 90 min was taken on the basis of current literature showing that SAHA in the range of 1 to 5 µM increases histone acetylation in an in vitro model within one hour, reaching the plateau effect at 3 h [31]. Therefore, we assumed that 90 min are sufficient to achieve the acetylation increase of all the other HDAC potential targets, including cytoplasmic substrates and to preserve cell viability. We demonstrated higher levels of SERCA2 acetylation following SAHA treatment in adult CMs from CTR rats by immunoprecipitation and Western blot for Acetylated-Lysine. The SERCA2 ATPase activity has been then explored using isolated microsomes from normal adult rat CMs, where an increase has been shown after SAHA treatment. Although it is a common praxis to perform measures at pCa5 [21,32,33,34], we decided to test the calcium-dependence of ATPase activity in order to confirm our results on SERCA2 ATPase activity also using microsomes isolated from HL-1 cells, as additional in vitro model. Specifically, HL-1 cells, derived from mouse atrial cardiomyocyte tumor lineage [22], represent a more abundant source of cardiomyocytes compared to freshly isolated rat adult CMs.

The treatment with SAHA ameliorated intracellular Ca^2+^ dynamics and, accordingly, contractile performance of adult rat CMs. These findings support the hypothesis that the inhibition of SERCA2 deacetylation induced by SAHA plays a crucial role in promoting a more efficient SR calcium re-uptake, which in turn results in a higher rate of re-lengthening and shorter relaxation times [35].

In view of these results, one should also consider that Gupta and colleagues recently observed an enhanced calcium sensitivity of myofilaments following HDAC inhibition. This aspect can play a key role in explaining the improved contractile efficiency of SAHA treated CMs [36]. On the other hand, an increase of myofilament calcium sensitivity would be expected to delay SR Ca^2+^ uptake and consequently prolong twitch duration, thus having an opposite effect compared to our observation. Nevertheless, it is important to notice that Gupta and coworkers have demonstrated that acetylation increases myofilament Ca^2+^ sensitivity in a model of skinned cardiac papillary muscle fibers [36]. It is known that the muscle skinning process leads to the loss of all the other sensitizing and desensitizing intracellular components that synergistically act in determining the effective calcium sensitivity of myofilaments [37]. Therefore it is conceivable that the discrepancy between our and Gupta results depends, at least in part, on the use of a whole cell model instead of a reductionist model consisting only of free calcium ions and myofilaments.

It is now well recognized that Ca^2+^ handling not only governs contractile events, but can also directly or indirectly influence ion channel function in cardiomyocytes, resulting in electrophysiological effects. An important component of Na^+^ channel regulation is due to Ca^2+^, calmodulin (CaM) and CaM-dependent protein kinase II (CaMKII) pathway that affects channel function [38]. L-type Ca^2+^ (*I*CaL) and sodium(Na^+^)-Ca^2+^ exchanger (NCX) currents are Ca^2+^-sensitive, as well as the slowly activating delayed rectifier current (*I*Ks), which plays an important role in regulating action potential duration [39,40]. In this work, we did not perform electrophysiological measurements on isolated ventricular myocytes that could help us in evaluating the impact on cardiac ion channels and pumps of SERCA2a activity amelioration induced by SAHA deacetylation inhibition. However, several findings reported in the present study can be useful to speculate on this topic. We observed that the amplitude of the calcium transient, which is dependent on the amplitude of Ca^2+^ entry, was comparable in control and SAHA-treated cardiomyocytes, suggesting that SAHA does not induce substantial changes in the calcium entry, through L-type Ca^2+^ channels and reverse activity of NCX. In addition, ryanodine receptors should not be affected by SAHA treatment, as indirectly suggested by the calcium transient rate of rise, measured as time to peak fluorescence, showing similar values in treated and untreated cells. Also, it should be considered that diastolic and systolic cytosolic Ca^2+^ levels were unchanged in SAHA treated cells as compared to control cardiomyocytes. This observation, besides confirming that calcium homeostasis is maintained even in the presence of a faster SERCA2 calcium re-uptake, should imply no changes in both the intracellular Na^+^ concentration that is tightly coupled to calcium concentration regulation, via electrogenic Na^+^/Ca^2+^ exchange, and Ca^2+^-sensitive *I*Ks current.

Interestingly, it has been recently shown that SAHA administration in vivo in a mouse model of muscular dystrophy can reduce the appearance of cardiac arrhythmias by reverting the remodeling of connexins and sodium channels [17]. Although the mechanisms of action are still unclear, those findings, together with our results, support the positive effect of SAHA treatment on cardiac function.

In order to explore possible acetylation sites influencing human SERCA2 activity, 3D models of hSERCA2a were produced by bioinformatic prediction methods, identifying the residue 464 as the most promising candidate. Residue K464 is located at the surface of the N domain in hSERCA2a. Although homology modeling of the different conformational states does not reveal an obvious functional impact for the K464 acetylation, the site is expected to locate on the side of a large cleft in state E2P [1,41] at the interface between domains N, P and M, near a putative phospholamban binding site (Figure S4). The potential effect of the K464 acetylation in protein electrostatics, flexibility and phospholamban binding affinity should be further investigated. Indeed, this lysine, conserved in all mammals, has already been reported as acetylated in the guinea pig heart, although no evidence of its functional role has been provided [15].

We produced three mutant HEK lines expressing WT hSERCA2a, or hSERCA2a where K464 was mutated into Q or R, mimicking constitutive acetylated and deacetylated state of the residue, respectively [15]. Of note, HEK cells have already been used by other groups as a valuable model to overexpress mutant SERCA and to study the consequent effects on the protein pumping activity [11]. HEK cells overexpressing mutated SERCA have also been recently used as a tool to investigate intracellular calcium dynamics [42]. Importantly, despite the debate on the presence of functional RyRs in HEK cells [43,44,45], we found that the application of a pulse of caffeine caused the abrupt increase of intracellular calcium concentration. Therefore, we decided to expose HEK cells transfected with WT hSERCA2a or mutated hSERCA2a to caffeine in order to increase the intracellular calcium concentration and then compare the time of recovery in WT compared to mutants. It is important to notice that, although the recovery phase in HEK cells was considerably longer than that observed in adult cardiomyocytes, SERCA has been described as the major player in the recovery of calcium transients long up to 4 s [46]. We cannot exclude a possible involvement of other Ca^2+^ removal systems (such as mitochondria and Peripheral Membrane Calcium ATPase) as well as of Ca^2+^ buffering in restoring basal conditions. However, given that in our experimental conditions the hSERCA2a pump was overexpressed, we can reasonably assume that the recovery phase in (transfected) HEK cells at least partially depends on the activity of transfected hSERCA2a. Intriguingly, K464Q mutants exhibited a higher ATPase activity and an acceleration of Ca^2+^ transient recovery phase compared to WT, supporting the hypothesis that the acetylation is a positive regulator of SERCA2 function. However, it is important to notice that SAHA could inhibit the acetylation of different lysine residues at the same time. Thus, mutating only one lysine at a time might only partially mimic the global effect obtained after HDAC inhibition on CMs. 

Interestingly, Hajjar and coworkers demonstrated that sumoylation of lysine residue 585 enhances SERCA2a stability and activity [11]. It has been recently shown that HDAC inhibition can stimulate protein sumoylation possibly through acetylation of the SUMO machinery [47]. Thus, our findings in isolated SAHA-treated cardiomyocytes sustain the idea that HDAC-inhibition could support other approaches, such as SUMO-1 overexpression [11], in the treatment of heart disease associated with SERCA2 impairment.

The main limitation of the present study is the fact that acetylation is only one of the factors contributing to SERCA2 function in health and disease. Additionally, we cannot exclude a possible action of SAHA on other proteins, such as SERCA2 interacting proteins or transporters involved in calcium dynamics, such as the sodium-calcium exchanger [48]. However, SERCA2 is recognized as the main player in calcium re-uptake during the relaxation/recovery phase in CMs, removing approximately 70–90% of calcium ions from the cytosol in species including humans, mice and rats [4]. This observation let us hypothesize that SERCA2 is the key factor in SR reuptake whose function is modulated by SAHA. The fact that the time to peak (TTP) of calcium transients recorded in isolated rat cardiomyocytes remained unchanged following SAHA administration seems also to indicate that RyRs are not affected by the treatment, at least under our experimental conditions. Further, our results indicate that phosphorylated and total PLB remain unchanged after treatment, thus suggesting that PKA activity might not be affected by SAHA. Nevertheless, deeper investigation are required in order to explain whether acetylation can directly or indirectly modulate other mechanisms of SERCA2 regulation, like CAMKII mediated phosphorylation. Importantly, it has been recently hypothesized that SERCA2 can be potentially regulated by microRNAs [49]. Concomitantly, experimental evidence has been provided that knock-out mice for miR-22 expression exhibit prolonged calcium transients and a decrease of SERCA transcript induced by indirect interaction [50]. More recently, it has been shown that miR-275 can directly target SERCA in mosquito gut [51], thus providing new basis for further studies on SERCA regulation by microRNA in vertebrates and humans. In this context, it should be reported that SAHA is well recognized as a microRNA modulator [52,53,54]. Further studies are required to understand whether HDAC inhibition can exert its action on SERCA and calcium dynamics by acting on microRNA target. Additionally, other potential mechanism of action should be taken into consideration for future studies, such as the role of the TGF-beta pathway, already shown to be involved in cardiac calcium signaling [55,56] and to be modulated by HDAC inhibitors in organs other than heart [57,58,59].

However, we would like to underline the fact that the aim of the present paper was to evaluate whether HDAC inhibition can modulate cardiac cell function by acting on SERCA2. SERCA2 is reasonably one of the multiple targets of SAHA action that can result in the enhancement or in the inhibition of many proteins, the net effect on EC coupling depending on the combination of the single contributions and on the model used.

Another question remaining unanswered is which enzymes are responsible for SERCA2a acetylation/deacetylation. It is known that SAHA at low concentration is a potent inhibitor of the class I HDAC isoforms 1 and 8 (IC50 < 20 nM), while it is active on other class I and class II HDACs at higher concentrations [60]. It is also well recognized that class II HDAC have both nuclear and cytoplasmic localization [61]. However, although traditionally considered as nuclear enzymes, class I HDACs have been recently demonstrated to be present in the endoplasmic reticulum [62]. Therefore, it is conceivable that both class of enzymes might act on SERCA2, contributing to the control of its acetylation status. Further studies are required to elucidate this point.

In conclusion, the results here demonstrate for the first time that SERCA2 acetylation and activity are increased following HDAC inhibition promoted by SAHA. Importantly, our findings suggest that the modulation of acetylation can be a novel strategy to treat diseases where calcium handling is affected, such as diabetic cardiomyopathy [6,63], dilated cardiomyopathy [64], ischemia/reperfusion injury [65], cardiac hypertrophy [66] and heart failure [67].

## 4. Materials and Methods

### 4.1. Rat Population

This study was carried out in strict accordance with the recommendations in the Guide for the Care and Use of Laboratory Animals (National Institute of Health, Bethesda, MD, USA, revised 1996)*.* The investigation was approved by the Veterinary Animal Care and Use Committee of the University of Parma-Italy (Prot. No. 59/12; 9 May 2012) and conforms to the National Ethical Guidelines of the Italian Ministry of Health. All efforts were made to minimize animal suffering.

The study population consisted of 5 male Wistar rats (*Rattus norvegicus*) aged 12–14 weeks, weighing 379 ± 8 g, individually housed in a temperature-controlled room at 22–24 °C, with the light on between 7.00 AM and 7.00 PM.

### 4.2. Rat Cardiomyocyte Isolation

Individual left ventricular (LV) cardiomyocytes (CMs) were enzymatically isolated by collagenase perfusion from the adult rat hearts in accordance with a procedure previously described [68]. Briefly, rats were anesthetised with ketamine chloride (Imalgene, Merial, Milan, Italy; 40 mg/kg ip) plus medetomidine hydrochloride (Domitor, Pfizer Italia S.r.l., Latina, Italy; 0.15 mg/kg ip). The heart was then removed and rapidly perfused at 37 °C by means of an aortic cannula with the following sequence of solutions: (1) a calcium-free solution for 5 min to remove the blood. Calcium-free solution contains the following (in mM): 126 NaCl, 22 dextrose, 5 MgCl_2_, 4.4 KCl, 20 taurine, 5 creatine, 5 sodium pyruvate, 1 NaH_2_PO_4_, and 24 HEPES (pH 7.4, adjusted with NaOH), and the solution was gassed with 100% O_2_; (2) a low-calcium solution (0.1 mM) plus 1 mg/mL type 2 collagenase (Worthington Biochemical, NJ, USA) and 0.1 mg/mL type XIV protease (Sigma-Aldrich, Milan, Italy) for about 20 min; (3) an enzyme-free, low-calcium solution for 5 min. The left ventricle was then minced and shaken for 10 min. The cells were filtered through a nylon mesh, re-suspended in low-calcium solutions (0.1 mM) for 30 min, and then used for recording cell mechanics and calcium transients. Following enzymatic dissociation, CMs were either untreated or incubated with 2.5 µM SAHA for 90 min. A fraction of cells from each experimental group was washed three times with low-calcium solution and centrifuged (42× *g* for 5 min). After removing the supernatant, the pellet was stored at −80 °C for subsequent microsome isolation (see the Section 4.6). The remaining cardiomyocytes were used for recording cell mechanics and calcium transients, as described in the Section 4.8.

### 4.3. HL-1 Cardiomyocyte Culture

HL-1 cells, derived from the AT-1 mouse atrial cardiomyocyte tumor lineage [22], were kindly donated by Prof. W.C. Claycomb. Cells were grown as previously described [22] in Claycomb Medium (Sigma-Aldrich S.r.l., Milan, Italy) supplemented with 100 μM norepinephrine (Sigma-Aldrich), 10% FBS (Sigma-Aldrich), 1% *v*/*v* penicillin streptomycin (Invitrogen by Thermo Fisher Scientific, Monza, Italy) and 2 mM l-glutamine (Invitrogen). Cells were cultured at 37 °C in a humid atmosphere of 5% CO_2_ and 95% air. Cells were plated onto gelatin/fibronectin-coated flasks at a density of 10,000 cells/cm^2^. When cells reached 90% of confluency, culture medium was replaced and supplemented for 90 min with 2.5 µM SAHA. 

### 4.4. Western Blot Analysis

Cells were washed in PBS and harvested in protein extraction buffer (10 mM Tris-HCl pH 7.4, 150 mM NaCl, 1% Igepal CA630, 1% sodium deoxycholate, 0.1% SDS (Sodium Dodecyl Sulphate) and 1% Glycerol supplemented with protease/phosphatase inhibitor mix (Roche Diagnostics S.p.A., Milan, Italy). Proteins were quantified by BCA protein kit (Thermo Fisher Scientific, Monza, Italy) following manufacturer’s instructions. Then, 30 µg of protein extracts were separated by SDS-PAGE on precast gradient (4–12%) gels (Invitrogen) using MOPS running buffer (Invitrogen) and transferred onto nitrocellulose membranes (Bio-Rad, Segrate, Milan, Italy) in Transfer buffer (Life Technologies by Thermo Fisher Scientific) supplemented with 10% (*v*/*v*) methanol (Sigma-Aldrich, Milan, Italy). After blocking in 5% BSA in PBS containing 0.05% Tween 20 (1 h at room temperature), membranes were incubated overnight at 4 °C with primary antibodies: SERCA2 (1:1000, SantaCruz, Dallas, TX, USA, sc-376235), Acetylated-tubulin (1:1000, Sigma-Aldrich # T7451), GAPDH (glyceraldehyde-3-phosphate dehydrogenase, 1:2000, SantaCruz, Dallas, TX, USA, sc-32233), Pan-Ac-K (Acetylated-Lysine, 1:1000, Cell Signaling, Danvers, MA, USA, #9441), Phospho-PLB (Ser16) (Phospho-Phospholamban (Ser16), 1:5000, Merck Millipore, Billerica, MA, USA, # 07-052) and PLB (Phospholamban, 1:8000, Abcam, Hong Kong, China, #ab2865). Blots were washed three times in PBS-Tween buffer and then incubated with appropriate horseradish peroxidase conjugated secondary antibody (SantaCruz) for 1 h at room temperature. Detection was performed by enhanced chemiluminescence system (Supersignal West Dura Extended Duration Substrate, Thermo Scientific). Results were quantified by Image Lab software 5.2.1 (BioRad, Segrate-Milan, Italy).

### 4.5. Immunoprecipitation

Co-immunoprecipitation experiments were performed using protein-Agarose accordingly to the manufacturer’s protocol (Roche Diagnostics S.p.A., Milan, Italy). In particular, 1 mg of protein extracts was co-immunoprecipitated with 10 µg of anti-SERCA2 antibody (SantaCruz, Dallas, TX, USA). Negative controls were performed with the same amount of protein extract (derived from rat cardiomyocytes) and were immunoprecipitated with the corresponding purified IgG antisera (SantaCruz) in the absence of primary antibody. Immunoprecipitated samples were resolved by SDS–PAGE, transferred onto nitrocellulose membrane (Bio-Rad, Segrate-Milan; Italy) and western blots were performed as described in the Section 4.4.

### 4.6. Isolation of the Microsomal Fraction

Microsome isolation from rat cardiomyocytes and HL-1 cells was performed according to Maruyama and MacLennan (1988) with minor modifications [69]. Specifically, cells were washed twice with PBS and then homogenized with 30 strokes in a glass Dounce homogenizer in 10 mM Tris-HCl, pH 7.5, 0.5 mM MgCl_2_. The homogenate was diluted with an equal volume of a solution of 10 mM Tris-HCl, pH 7.5, 0.5 M sucrose, 300 mM KCl. The suspension was centrifuged at 10,000× *g* for 30 min at 4 °C to pellet nuclei and mitochondria. The pellet was discarded and the supernatant was further centrifuged at 100,000× *g* for 150 min at 4 °C to sediment the microsomal fraction. The pellet was re-suspended in a solution containing 0.25 M sucrose and 10 mM MOPS. All solutions were enriched in protease inhibitors (Roche). The microsome concentration was measured using the BCA protein kit (Thermo Fisher Scientific) following the manufacturer’s instructions.

### 4.7. ATP/NADH Coupled Assay for Calcium ATPase Activity

The ATPase activity of the microsomal fraction isolated from HL-1 cells (25 μg/mL), was measured by spectrophotometric determination of NADH oxidation coupled to an ATP regenerating system, as previously described [70]. We used a Beckman DU 640 spectrophotometer adjusted at a wavelength of 340 nm. The assay was performed at 37 °C in a final volume of 1 mL of a buffer containing 20 mM histidine pH 7.2, 5 mM MgCl_2_, 0.5 mM EGTA, 100 mM KCl, 2 mM ATP, 0.5 mM phosphoenolpyruvate, 0.15 mM NADH, 1.4 units of pyruvate kinase/lactic dehydrogenase, in the presence of 2 μg/mL A23187 Ca^2+^-ionophore. ATPase activity was obtained subtracting the basal activity (measured in the presence of EGTA without calcium added) from the maximal activity and was expressed as micromoles/min/mg of protein. For investigating the calcium-dependence of ATPase activity, the concentration of free calcium was varied from pCa8 to pCa5 using EGTA buffered solutions. 

NADH coupled ATPase assay protocol was then adapted for use on a 96-well microplate reader, according to the method described by Kiianitsa and coworkers [71]. We used the microplate protocol to analyze the ATPase activity of the microsomal fraction isolated from adult rat ventricular cardiomyocytes (5 μg/well) and from HEK293 cells (20 μg/well) expressing either Wild Type or mutated human SERCA2a (see Section 4.10). The absorbance change at 340 nm was monitored using the EnVision (Perkin Elmer, Waltham, MA, USA) plate reader. The experiments were performed at 37 °C in a final volume of 200 μL at pCa5 [21] on the same buffer described above. Technical duplicates were performed for each experiment.

### 4.8. Rat Cardiomyocyte Mechanics and Calcium Transients

CM mechanical properties were assessed by using the IonOptix fluorescence and contractility systems (IonOptix, Milton, MA, USA). CMs were placed in a chamber mounted on the stage of an inverted microscope (Nikon-Eclipse TE2000-U, Nikon Instruments, Florence, Italy) and superfused (1 mL/min at 37 °C) with a Tyrode solution containing (in mM): 140 NaCl, 5.4 KCl, 1 MgCl_2_, 5 HEPES, 5.5 glucose, and 1 CaCl_2_ (pH 7.4, adjusted with NaOH). Only rod-shaped myocytes with clear edges and average sarcomere length ≥1.7 µm were selected for the analysis. All the selected myocytes did not show spontaneous contractions. The cells were field stimulated at a frequency of 0.5 Hz by constant current pulses (2 ms in duration, and twice diastolic threshold in intensity) delivered by platinum electrodes placed on opposite sides of the chamber, connected to a MyoPacer Field Stimulator (IonOptix). The stimulated myocyte was displayed on a computer monitor using an IonOptix MyoCam camera. Load-free contraction of myocytes was measured with the IonOptix system, which captures sarcomere length dynamics via a Fast Fourier Transform algorithm. Sampling rate was fixed at 1 KHz. A total of 115 control isolated ventricular myocytes (of which 45 were used as untreated control and 70 were exposed to SAHA) were analyzed to compute the following parameters: mean diastolic sarcomere length and fraction of shortening (FS), maximal rates of shortening and re-lengthening (±dl/dt_max_), and time at 10%, 50% and 90% of re-lengthening (RL10, RL50 and RL90, respectively). Steady-state contraction of myocytes was achieved before data recording by means of a 10 s conditioning stimulation.

Calcium transients were measured simultaneously with cell motion. Ca^2+^ transients were detected by epifluorescence after loading the myocytes with Fluo-3 AM (10 µmol/L; Invitrogen, Carlsbad, CA, USA) for 30 min. Excitation length was 480 nm, with emission collected at 535 nm using a 40× oil objective lens (NA: 1.3). Fluo-3 signals were expressed as normalized fluorescence (f/f0: fold increase). The time course of the fluorescence signal decay was described by a single exponential equation, and the time constant (tau) was used as a measure of the rate of intracellular calcium clearing [72]. The time to peak of the calcium transients (TTP) and the times to 10%, 50% and 90% of fluorescence signal decay were also measured (time to BaseLine fluorescence BL10, BL50 and BL90, respectively).

### 4.9. Bioinformatics Analysis

The post-translation modification databases PHOSIDA [73] and PhosphoSitePlus [74] were used for the identification of acetylation sites investigated in human SERCA2. The location of the lysine acetylation was investigated in human SERCA2a structural models derived by homology modelling based on rabbit homologue templates with 83% sequence identity to hSERCA2a, and using MODELLER version 9.11 [75].

### 4.10. Mutagenesis

SERCA2a mutants were generated by PCR-site directed mutagenesis (for a list of primers see Table S1) and verified by complete sequencing (Eurofins Genomics, Ebersberg, Germany). The SERCA2a wild-type coding sequence (NM_001681.3; SER-WT) as well as the SERCA2a coding sequence with Lys 464 residue mutated into glutamine (SER-K464Q) or arginine (SER-K464R) were subsequently cloned into the pCDNA3 vector carrying Geneticin resistance (Life Technologies) for subsequent cell transfection. Transfections were performed on HEK-MSR1 cells (Human Embryonic Kidney 293 cells) that stably express human Macrophage Scavenger Receptor 1, facilitating cell adhesion in blasticidin selection (5 µg/mL, Invitrogen) [76]. Lipofectamine LTX (Invitrogen) was used for transfection following manufacturer’s instructions. Stable cell lines were selected 24 h after transfections in culture medium composed by: MEM (Invitrogen) supplemented with 10% FBS (Sigma-Aldrich), 1% *v*/*v* glutamine (Invitrogen), 1% *v*/*v* minimum non-essential amino acids (Invitrogen) and 450 µg/mL Geneticin (Invitrogen).

#### 4.1.1. SERCA2 Mutant Calcium Transients

HEK-MSR1 stably transfected with K464 onto SERCA2 sequence mutated in Q or R were plated on the bottom of glass dishes (25 mm diameter) and grown for 2 days in MEM medium supplemented with FBS 10%. Cytosolic Ca^2+^ dynamics were evaluated in Fluo-4 AM loaded cells. Briefly, cells were loaded with 2.5 µM of Fluo-4 AM (Molecular Probes, Invitrogen) for 30 min at 37 °C and then washed with Tyrode’s solution containing in mM: 10 d-(+)-glucose, 5.0 Hepes, 140 NaCl, 5.4 KCl, 1.2 MgCl_2_, 1.8 CaCl_2_, pH adjusted to 7.3 with NaOH. Dishes were mounted in a perfusion chamber and placed on the stage of an epifluorescence microscope (Nikon) equipped with a 75 W Xenon lamp and connected to a CCD camera (Cool SnapTM EZ Photometrics, Tucson, AZ, USA). Cells were excited at 488 nm wavelength, fluorescence emission was measured at 520 nm and recorded using the MetaFluor Software (Molecular Devices, Sunnyvale, CA, USA)*;* imaging was scanned repeatedly at 20 Hz. This low temporal acquisition is justified by the longer calcium transient in HEK cells lasting on average 1 min and 20 s. Calcium transients were evoked by a 1 s caffeine-pulse (10 mM). Temperature was maintained at 36.5 ± 0.5 °C throughout the experiment.

The background fluorescence signal was fitted to a linear regression and then subtracted. Each trace was subsequently resampled and denoised with an Rbf interpolator (f = 20, e = 1, s = 1). The baseline was calculated per-trace using the median value of the first 250 ms before the excitation and then subtracted.

Traces which didn’t reach a peak value of at least 2 times the baseline level, or dropped under 90% of the absolute value of the baseline were discarded. We then calculated the time to decay at the first intersection of 10%, 50% and 90% of the curves, thus defining time to BL10, BL50 and BL90. Only traces that successfully recovered to BL90 within the recording timeline (90 s) were considered.

#### 4.1.2. Statistical Analysis

All data are reported as means ± standard error (SEM). Normality of the data was evaluated by D’Agostino & Pearson normality test. For two groups, significance was assessed by two-tailed unpaired *t*-test or non-parametric Mann–Whitney *U*-test when appropriate. For more than two groups either two-way ANOVA, parametric one-way ANOVA or Kruskal-Wallis non parametric test were used followed by appropriate post-hoc individual comparisons (GraphPad Prism software 6.03). General Linear Model (GLM) ANOVA for repeated measurements was used to compare cell contractility and calcium transient data (IBM-SPSS 24.0, SPSS Inc., Chicago, IL, USA). The details on the specific test used are reported in the figure legend of each specific experiment. A *p*-value < 0.05 was considered statistically significant.

## Supplementary Materials

Supplementary materials can be found at http://www.mdpi.com/1422-0067/19/2/419/s1.

## References

[1] Toyoshima C. (2008). Structural aspects of ion pumping by Ca^2+^-ATPase of sarcoplasmic reticulum. Arch. Biochem. Biophys..

[2] Asahi M., Sugita Y., Kurzydlowski K., De Leon S., Tada M., Toyoshima C., MacLennan D.H. (2003). Sarcolipin regulates sarco(endo)plasmic reticulum Ca^2+^-ATPase (SERCA) by binding to transmembrane helices alone or in association with phospholamban. Proc. Natl. Acad. Sci. USA.

[3] Periasamy M., Kalyanasundaram A. (2007). SERCA pump isoforms: Their role in calcium transport and disease. Muscle Nerve.

[4] Bers D.M. (2002). Cardiac excitation-contraction coupling. Nature.

[5] Sulaiman M., Matta M.J., Sunderesan N.R., Gupta M.P., Periasamy M., Gupta M. (2010). Resveratrol, an activator of SIRT1, upregulates sarcoplasmic calcium ATPase and improves cardiac function in diabetic cardiomyopathy. Am. J. Physiol. Heart Circ. Physiol..

[6] Savi M., Bocchi L., Mena P., Dall’Asta M., Crozier A., Brighenti F., Stilli D., Del Rio D. (2017). In vivo administration of urolithin A and B prevents the occurrence of cardiac dysfunction in streptozotocin-induced diabetic rats. Cardiovasc. Diabetol..

[7] Del Monte F., Harding S.E., Schmidt U., Matsui T., Kang Z.B., Dec G.W., Gwathmey J.K., Rosenzweig A., Hajjar R.J. (1999). Restoration of contractile function in isolated cardiomyocytes from failing human hearts by gene transfer of SERCA2a. Circulation.

[8] Park W.J., Oh J.G. (2013). SERCA2a: A prime target for modulation of cardiac contractility during heart failure. BMB Rep..

[9] Periasamy M., Bhupathy P., Babu G.J. (2008). Regulation of sarcoplasmic reticulum Ca^2+^ ATPase pump expression and its relevance to cardiac muscle physiology and pathology. Cardiovasc. Res..

[10] Stammers A.N., Susser S.E., Hamm N.C., Hlynsky M.W., Kimber D.E., Kehler D.S., Duhamel T.A. (2015). The regulation of sarco(endo)plasmic reticulum calcium-ATPases (SERCA). Can. J. Physiol. Pharmacol..

[11] Kho C., Lee A., Jeong D., Oh J.G., Chaanine A.H., Kizana E., Park W.J., Hajjar R.J. (2011). SUMO1-dependent modulation of SERCA2a in heart failure. Nature.

[12] Adachi T., Weisbrod R.M., Pimentel D.R., Ying J., Sharov V.S., Schoneich C., Cohen R.A. (2004). S-Glutathiolation by peroxynitrite activates SERCA during arterial relaxation by nitric oxide. Nat. Med..

[13] Bidasee K.R., Zhang Y., Shao C.H., Wang M., Patel K.P., Dincer U.D., Besch H.R. (2004). Diabetes increases formation of advanced glycation end products on Sarco(endo)plasmic reticulum Ca^2+^-ATPase. Diabetes.

[14] Knyushko T.V., Sharov V.S., Williams T.D., Schoneich C., Bigelow D.J. (2005). 3-Nitrotyrosine modification of SERCA2a in the aging heart: A distinct signature of the cellular redox environment. Biochemistry.

[15] Foster D.B., Liu T., Rucker J., O’Meally R.N., Devine L.R., Cole R.N., O’Rourke B. (2013). The cardiac acetyl-lysine proteome. PLoS ONE.

[16] Minucci S., Pelicci P.G. (2006). Histone deacetylase inhibitors and the promise of epigenetic (and more) treatments for cancer. Nat. Rev. Cancer.

[17] Colussi C., Berni R., Rosati J., Straino S., Vitale S., Spallotta F., Baruffi S., Bocchi L., Delucchi F., Rossi S. (2010). The histone deacetylase inhibitor suberoylanilide hydroxamic acid reduces cardiac arrhythmias in dystrophic mice. Cardiovasc. Res..

[18] McLendon P.M., Ferguson B.S., Osinska H., Bhuiyan M.S., James J., McKinsey T.A., Robbins J. (2014). Tubulin hyperacetylation is adaptive in cardiac proteotoxicity by promoting autophagy. Proc. Natl. Acad. Sci. USA.

[19] Bianchini E., Testoni S., Gentile A., Cali T., Ottolini D., Villa A., Brini M., Betto R., Mascarello F., Nissen P. (2014). Inhibition of ubiquitin proteasome system rescues the defective sarco(endo)plasmic reticulum Ca^2+^-ATPase (SERCA1) protein causing Chianina cattle pseudomyotonia. J. Biol. Chem..

[20] Lytton J., Westlin M., Burk S.E., Shull G.E., MacLennan D.H. (1992). Functional comparisons between isoforms of the sarcoplasmic or endoplasmic reticulum family of calcium pumps. J. Biol. Chem..

[21] Kho C., Lee A., Jeong D., Oh J.G., Gorski P.A., Fish K., Sanchez R., DeVita R.J., Christensen G., Dahl R. (2015). Small-molecule activation of SERCA2a SUMOylation for the treatment of heart failure. Nat. Commun..

[22] Claycomb W.C., Lanson N.A., Stallworth B.S., Egeland D.B., Delcarpio J.B., Bahinski A., Izzo N.J. (1998). HL-1 cells: A cardiac muscle cell line that contracts and retains phenotypic characteristics of the adult cardiomyocyte. Proc. Natl. Acad. Sci. USA.

[23] Mertins P., Qiao J.W., Patel J., Udeshi N.D., Clauser K.R., Mani D.R., Burgess M.W., Gillette M.A., Jaffe J.D., Carr S.A. (2013). Integrated proteomic analysis of post-translational modifications by serial enrichment. Nat. Methods.

[24] Choudhary C., Kumar C., Gnad F., Nielsen M.L., Rehman M., Walther T.C., Olsen J.V., Mann M. (2009). Lysine acetylation targets protein complexes and co-regulates major cellular functions. Science.

[25] Lytton J., Westlin M., Hanley M.R. (1991). Thapsigargin inhibits the sarcoplasmic or endoplasmic reticulum Ca-ATPase family of calcium pumps. J. Biol. Chem..

[26] Munshi A., Tanaka T., Hobbs M.L., Tucker S.L., Richon V.M., Meyn R.E. (2006). Vorinostat, a histone deacetylase inhibitor, enhances the response of human tumor cells to ionizing radiation through prolongation of gamma-H2AX foci. Mol. Cancer Ther..

[27] Witt O., Milde T., Deubzer H.E., Oehme I., Witt R., Kulozik A., Eisenmenger A., Abel U., Karapanagiotou-Schenkel I. (2012). Phase I/II intra-patient dose escalation study of vorinostat in children with relapsed solid tumor, lymphoma or leukemia. Klinische Pädiatrie.

[28] Butler L.M., Zhou X., Xu W.S., Scher H.I., Rifkind R.A., Marks P.A., Richon V.M. (2002). The histone deacetylase inhibitor SAHA arrests cancer cell growth, up-regulates thioredoxin-binding protein-2, and down-regulates thioredoxin. Proc. Natl. Acad. Sci. USA.

[29] Richon V.M., Sandhoff T.W., Rifkind R.A., Marks P.A. (2000). Histone deacetylase inhibitor selectively induces p21WAF1 expression and gene-associated histone acetylation. Proc. Natl. Acad. Sci. USA.

[30] Xie M., Kong Y., Tan W., May H., Battiprolu P.K., Pedrozo Z., Wang Z.V., Morales C., Luo X., Cho G. (2014). Histone deacetylase inhibition blunts ischemia/reperfusion injury by inducing cardiomyocyte autophagy. Circulation.

[31] Tiffon C., Adams J., van der Fits L., Wen S., Townsend P., Ganesan A., Hodges E., Vermeer M., Packham G. (2011). The histone deacetylase inhibitors vorinostat and romidepsin downmodulate IL-10 expression in cutaneous T-cell lymphoma cells. Br. J. Pharmacol..

[32] Simonides W.S., van Hardeveld C. (1990). An assay for sarcoplasmic reticulum Ca^2+^-ATPase activity in muscle homogenates. Anal. Biochem..

[33] Kennedy D., Omran E., Periyasamy S.M., Nadoor J., Priyadarshi A., Willey J.C., Malhotra D., Xie Z., Shapiro J.I. (2003). Effect of chronic renal failure on cardiac contractile function, calcium cycling, and gene expression of proteins important for calcium homeostasis in the rat. J. Am. Soc. Nephrol..

[34] Kennedy D.J., Vetteth S., Xie M., Periyasamy S.M., Xie Z., Han C., Basrur V., Mutgi K., Fedorov V., Malhotra D. (2006). Ouabain decreases sarco(endo)plasmic reticulum calcium ATPase activity in rat hearts by a process involving protein oxidation. Am. J. Physiol. Heart Circ. Physiol..

[35] Frank K.F., Bolck B., Erdmann E., Schwinger R.H. (2003). Sarcoplasmic reticulum Ca^2+^-ATPase modulates cardiac contraction and relaxation. Cardiovasc. Res..

[36] Gupta M.P., Samant S.A., Smith S.H., Shroff S.G. (2008). HDAC4 and PCAF bind to cardiac sarcomeres and play a role in regulating myofilament contractile activity. J. Biol. Chem..

[37] Chung J.H., Biesiadecki B.J., Ziolo M.T., Davis J.P., Janssen P.M. (2016). Myofilament Calcium Sensitivity: Role in Regulation of In vivo Cardiac Contraction and Relaxation. Front. Physiol..

[38] Chen-Izu Y., Shaw R.M., Pitt G.S., Yarov-Yarovoy V., Sack J.T., Abriel H., Aldrich R.W., Belardinelli L., Cannell M.B., Catterall W.A. (2015). Na^+^ channel function, regulation, structure, trafficking and sequestration. J. Physiol..

[39] Kennedy M., Bers D.M., Chiamvimonvat N., Sato D. (2017). Dynamical effects of calcium-sensitive potassium currents on voltage and calcium alternans. J. Physiol..

[40] Bers D.M., Chen-Izu Y. (2015). Sodium and calcium regulation in cardiac myocytes: From molecules to heart failure and arrhythmia. J. Physiol..

[41] Olesen C., Picard M., Winther A.M., Gyrup C., Morth J.P., Oxvig C., Moller J.V., Nissen P. (2007). The structural basis of calcium transport by the calcium pump. Nature.

[42] Ying J., Tong X., Pimentel D.R., Weisbrod R.M., Trucillo M.P., Adachi T., Cohen R.A. (2007). Cysteine-674 of the sarco/endoplasmic reticulum calcium ATPase is required for the inhibition of cell migration by nitric oxide. Arterioscler. Thromb. Vasc. Biol..

[43] Tong J., Du G.G., Chen S.R., MacLennan D.H. (1999). HEK-293 cells possess a carbachol- and thapsigargin-sensitive intracellular Ca^2+^ store that is responsive to stop-flow medium changes and insensitive to caffeine and ryanodine. Biochem. J..

[44] Querfurth H.W., Haughey N.J., Greenway S.C., Yacono P.W., Golan D.E., Geiger J.D. (1998). Expression of ryanodine receptors in human embryonic kidney (HEK293) cells. Biochem. J..

[45] Luo D., Sun H., Xiao R.P., Han Q. (2005). Caffeine induced Ca^2+^ release and capacitative Ca^2+^ entry in human embryonic kidney (HEK293) cells. Eur. J. Pharmacol..

[46] Itzhaki I., Rapoport S., Huber I., Mizrahi I., Zwi-Dantsis L., Arbel G., Schiller J., Gepstein L. (2011). Calcium handling in human induced pluripotent stem cell derived cardiomyocytes. PLoS ONE.

[47] Blakeslee W.W., Wysoczynski C.L., Fritz K.S., Nyborg J.K., Churchill M.E., McKinsey T.A. (2014). Class I HDAC inhibition stimulates cardiac protein SUMOylation through a post-translational mechanism. Cell Signal..

[48] Bers D.M., Despa S. (2006). Cardiac myocytes Ca^2+^ and Na^+^ regulation in normal and failing hearts. J. Pharmacol. Sci..

[49] Bostjancic E., Zidar N., Glavac D. (2012). MicroRNAs and cardiac sarcoplasmic reticulum calcium ATPase-2 in human myocardial infarction: Expression and bioinformatic analysis. BMC Genom..

[50] Gurha P., Abreu-Goodger C., Wang T., Ramirez M.O., Drumond A.L., van Dongen S., Chen Y., Bartonicek N., Enright A.J., Lee B. (2012). Targeted deletion of microRNA-22 promotes stress-induced cardiac dilation and contractile dysfunction. Circulation.

[51] Zhao B., Lucas K.J., Saha T.T., Ha J., Ling L., Kokoza V.A., Roy S., Raikhel A.S. (2017). MicroRNA-275 targets sarco/endoplasmic reticulum Ca^2+^ adenosine triphosphatase (SERCA) to control key functions in the mosquito gut. PLoS Genet..

[52] Lee E.M., Shin S., Cha H.J., Yoon Y., Bae S., Jung J.H., Lee S.M., Lee S.J., Park I.C., Jin Y.W. (2009). Suberoylanilide hydroxamic acid (SAHA) changes microRNA expression profiles in A549 human non-small cell lung cancer cells. Int. J. Mol. Med..

[53] Yang H., Lan P., Hou Z., Guan Y., Zhang J., Xu W., Tian Z., Zhang C. (2015). Histone deacetylase inhibitor SAHA epigenetically regulates miR-17-92 cluster and MCM7 to upregulate MICA expression in hepatoma. Br. J. Cancer.

[54] Poddar S., Kesharwani D., Datta M. (2016). Histone deacetylase inhibition regulates miR-449a levels in skeletal muscle cells. Epigenetics.

[55] Li S., Li X., Zheng H., Xie B., Bidasee K.R., Rozanski G.J. (2008). Pro-oxidant effect of transforming growth factor-beta1 mediates contractile dysfunction in rat ventricular myocytes. Cardiovasc. Res..

[56] Mufti S., Wenzel S., Euler G., Piper H.M., Schluter K.D. (2008). Angiotensin II-dependent loss of cardiac function: Mechanisms and pharmacological targets attenuating this effect. J. Cell. Physiol..

[57] Ammanamanchi S., Brattain M.G. (2004). Restoration of transforming growth factor-beta signaling through receptor RI induction by histone deacetylase activity inhibition in breast cancer cells. J. Biol. Chem..

[58] Khan S., Jena G. (2014). Sodium butyrate, a HDAC inhibitor ameliorates eNOS, iNOS and TGF-beta1-induced fibrogenesis, apoptosis and DNA damage in the kidney of juvenile diabetic rats. Food Chem. Toxicol..

[59] Xie L., Santhoshkumar P., Reneker L.W., Sharma K.K. (2014). Histone deacetylase inhibitors trichostatin A and vorinostat inhibit TGFbeta2-induced lens epithelial-to-mesenchymal cell transition. Investig. Ophthalmol. Vis. Sci..

[60] Huber K., Doyon G., Plaks J., Fyne E., Mellors J.W., Sluis-Cremer N. (2011). Inhibitors of histone deacetylases: Correlation between isoform specificity and reactivation of HIV type 1 (HIV-1) from latently infected cells. J. Biol. Chem..

[61] Clocchiatti A., Florean C., Brancolini C. (2011). Class IIa HDACs: From important roles in differentiation to possible implications in tumourigenesis. J. Cell. Mol. Med..

[62] Kahali S., Sarcar B., Prabhu A., Seto E., Chinnaiyan P. (2012). Class I histone deacetylases localize to the endoplasmic reticulum and modulate the unfolded protein response. FASEB J..

[63] Ligeti L., Szenczi O., Prestia C.M., Szabo C., Horvath K., Marcsek Z.L., van Stiphout R.G., van Riel N.A., Op den Buijs J., Van der Vusse G.J. (2006). Altered calcium handling is an early sign of streptozotocin-induced diabetic cardiomyopathy. Int. J. Mol. Med..

[64] Pieske B., Kretschmann B., Meyer M., Holubarsch C., Weirich J., Posival H., Minami K., Just H., Hasenfuss G. (1995). Alterations in intracellular calcium handling associated with the inverse force-frequency relation in human dilated cardiomyopathy. Circulation.

[65] Saini H.K., Dhalla N.S. (2005). Defective calcium handling in cardiomyocytes isolated from hearts subjected to ischemia-reperfusion. Am. J. Physiol. Heart Circ. Physiol..

[66] Balke C.W., Shorofsky S.R. (1998). Alterations in calcium handling in cardiac hypertrophy and heart failure. Cardiovasc. Res..

[67] Luo M., Anderson M.E. (2013). Mechanisms of altered Ca^2+^ handling in heart failure. Circ. Res..

[68] Zaniboni M., Pollard A.E., Yang L., Spitzer K.W. (2000). Beat-to-beat repolarization variability in ventricular myocytes and its suppression by electrical coupling. Am. J. Physiol. Heart Circ. Physiol..

[69] Maruyama K., MacLennan D.H. (1988). Mutation of aspartic acid-351, lysine-352, and lysine-515 alters the Ca^2+^ transport activity of the Ca^2+^-ATPase expressed in COS-1 cells. Proc. Natl. Acad. Sci. USA.

[70] Sacchetto R., Testoni S., Gentile A., Damiani E., Rossi M., Liguori R., Drogemuller C., Mascarello F. (2009). A defective SERCA1 protein is responsible for congenital pseudomyotonia in Chianina cattle. Am. J. Pathol..

[71] Kiianitsa K., Solinger J.A., Heyer W.D. (2003). NADH-coupled microplate photometric assay for kinetic studies of ATP-hydrolyzing enzymes with low and high specific activities. Anal. Biochem..

[72] Bassani J.W., Bassani R.A., Bers D.M. (1994). Relaxation in rabbit and rat cardiac cells: Species-dependent differences in cellular mechanisms. J. Physiol..

[73] Gnad F., Gunawardena J., Mann M. (2011). PHOSIDA 2011: The posttranslational modification database. Nucleic Acids Res..

[74] Hornbeck P.V., Zhang B., Murray B., Kornhauser J.M., Latham V., Skrzypek E. (2015). PhosphoSitePlus, 2014: Mutations, PTMs and recalibrations. Nucleic Acids Res..

[75] Marti-Renom M.A., Stuart A.C., Fiser A., Sanchez R., Melo F., Sali A. (2000). Comparative protein structure modeling of genes and genomes. Annu. Rev. Biophys. Biomol. Struct..

[76] Robbins A.K., Horlick R.A. (1998). Macrophage scavenger receptor confers an adherent phenotype to cells in culture. Biotechniques.

