# Supplementary material for: HDAC Inhibition Improves the Sarcoendoplasmic Reticulum Ca2+-ATPase Activity in Cardiac Myocytes
Source: Int J Mol Sci. 2018 Jan 31;19(2):419. doi: 10.3390/ijms19020419 (PMC5855641; doi:10.3390/ijms19020419)
Supplement: Supplementary file 1 [file ijms-19-00419-s001.pdf]

SUPPLEMENTARY MATERIALS

HDAC inhibition improves the sarcoendoplasmic reticulum Ca^{2+}-ATPase activity in cardiac myocytes

Viviana Meraviglia, PhD[1†], Leonardo Bocchi, PhD[2†], Roberta Sacchetto, PhD [3], Maria Cristina Florio, PhD[1], Benedetta M. Motta, PhD[1], Corrado Corti, PhD [1], Christian X. Weichenberger, PhD[1], Monia Savi, PhD[2], Yuri D'Elia[1], Marcelo D. Rosato-Siri, PhD[1], Silvia Suffredini, PhD[1], Chiara Piubelli, PhD[1], Giulio Pompilio, MD[4, 5], Peter P. Pramstaller, MD[1], Francisco S. Domingues, PhD[1], Donatella Stilli, PhD[2‡], Alessandra Rossini, PhD[1‡].

SUPPLEMENTARY METHODS

Measurement of diastolic and systolic calcium concentration assessed by Fura-2 dye

Rat cardiomyocytes were loaded with Fura-2 by incubation of a 1 ml suspension of isolated cardiomyocytes for 10 min at room temperature with 2.0 µM Fura-2-acetoxymethyl ester (Invitrogen) plus Pluronic (0.04%; Sigma-Aldrich). Then, cardiomyocytes were washed twice with low calcium (0.1 mM) solution to remove the remaining Fura-2 and complete the de-esterification of intracellular AM ester.

The loaded cells were placed in a perfusion chamber on the stage of an inverted microscope (Olympus IX71) and intracellular Ca^{2+} transients were measured with contractility recording system (IonOptix LLC, Milton USA) at 0.5 Hz pacing rate (field stimulation). In order to record Ca^{2+} transients, cells were excited at 360/380 nm and emission was measured at 510 nm. The ratio of the fluorescence emitted by 360 nm and 380 nm excitation was used as an indicator of intracellular free Ca^{2+} (ΔF360/380). The Ionwizard software (IonOptix, USA) was used to capture the changes of Fura-2 fluorescence intensity. Then, the actual intracellular calcium ion concentration was computed by applying in vitro calibration, according with the manufacturer's instructions (ION-Optix).

Table S1: List of primers used in mutagenesis experiment

SERWT-F	AAAGGTACCATGATGGAGAACGCGCACACCAAGACG
SERWT-R	TTTGGGCCCTTACTCCAGTATTGCAGGTTCCAGGTAG
K464Q-F	GAAGGGTCTTTCT<u>CAG</u>ATAGAACGTGC
K464Q-R	GGCATTTGCACGTTCTAT<u>CTG</u>AGAAAGACC
K464R-F	GAAGGGTCTTTCT<u>AGA</u>ATAGAACGTGC
K464R-R	GGCATTTGCACGTTCTAT<u>TCT</u>AGAAAGACC

Figure S1

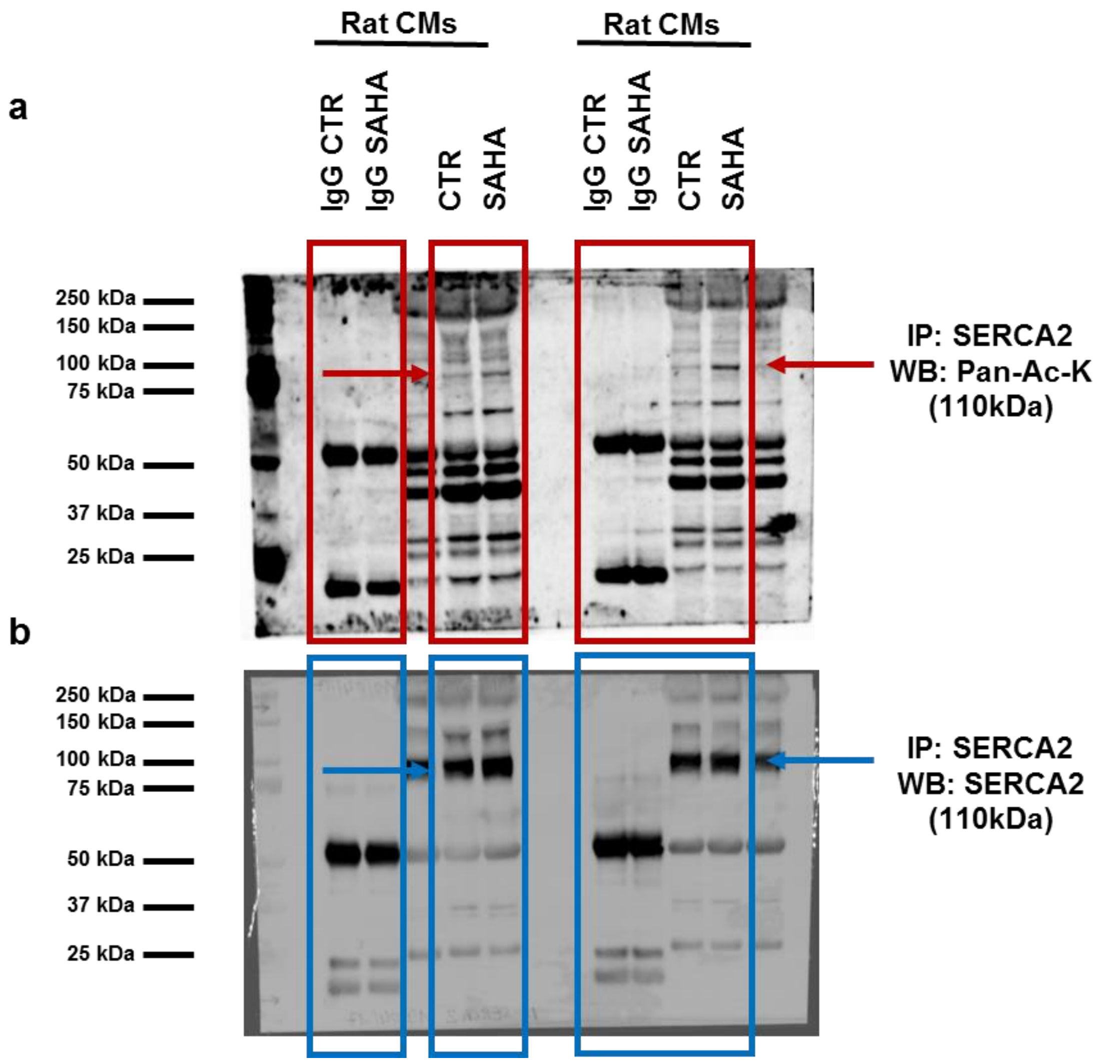

Figure S1. Representative examples of complete Western Blot panels showing immunoprecipitation experiments of SERCA2 on cardiomyocytes isolated from adult rat hearts after SAHA treatment. (**a**) The membrane displayed the Western blot with Pan-Ac-K antibody on lysates immunoprecipitated for SERCA2 protein. (**b**) The same membrane presented above showing the Western Blot with SERCA2 antibody on samples immunoprecipitated SERCA2. The acetylated bands in panel (**a**) (indicated by red arrows) corresponds to SERCA2 protein (indicated by blue arrows) as showed in panel (**b**).

Figure S2

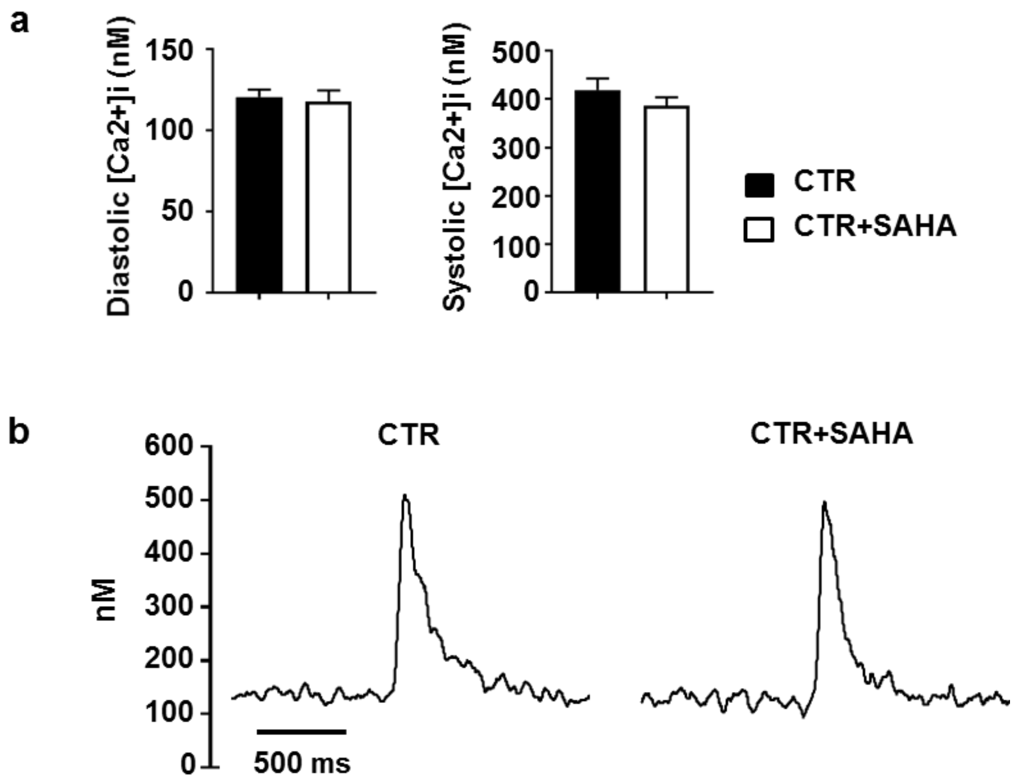

Figure S2. (**a**) Mean values±SEM of diastolic and systolic intracellular calcium concentration in control cardiomyocytes (CTR), and cardiomyocytes incubated with SAHA 2.5μM (CTR+SAHA). (**b**) Representative tracings of calcium transient recorded in the two groups of cells.

Figure S3

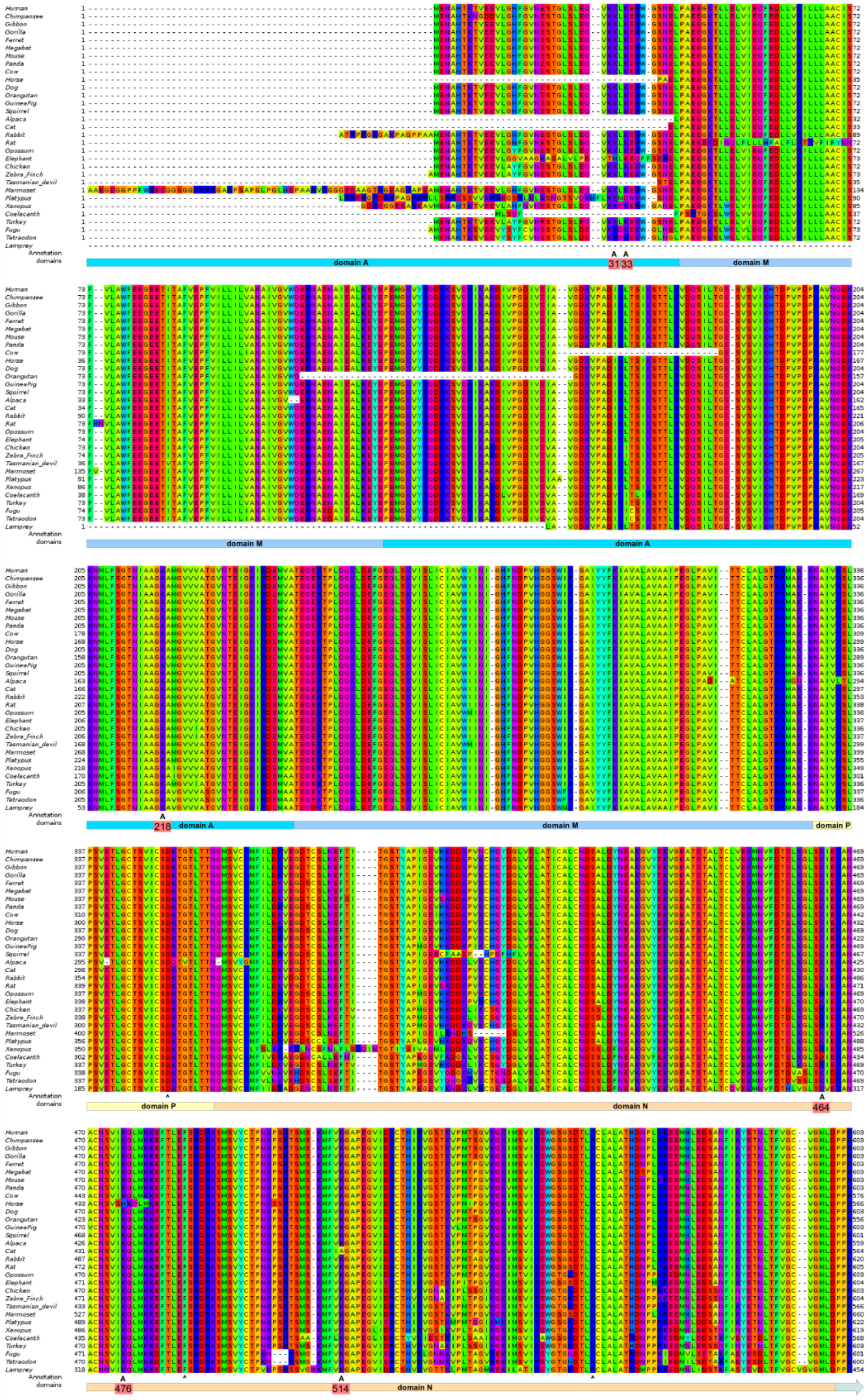

Figure S3. Multiple sequence alignment of SERCA2 orthologues. Alignment is shown up to the end of N domain. ADP binding residues marked with “^”. Acetylation sites according to PhosphoSitePlus are marked with “A”. Human SERCA2 acetylation sites are conserved in most mammals. SERCA2 orthologues were collected from Ensembl release 70 (http://www.ensembl.org/). Analysis was restricted to 1-to-1 orthologues. In addition, 14 orthologue sequences were excluded given extensive regions of unknown sequence, and two sequences were excluded because of problematic alignment. The sequences of human SERCA2a and respective orthologues were aligned with MUSCLE (http://www.drive5.com/muscle/), there was only a minor manual alignment correction at position 509 in the human sequence.

Figure S4

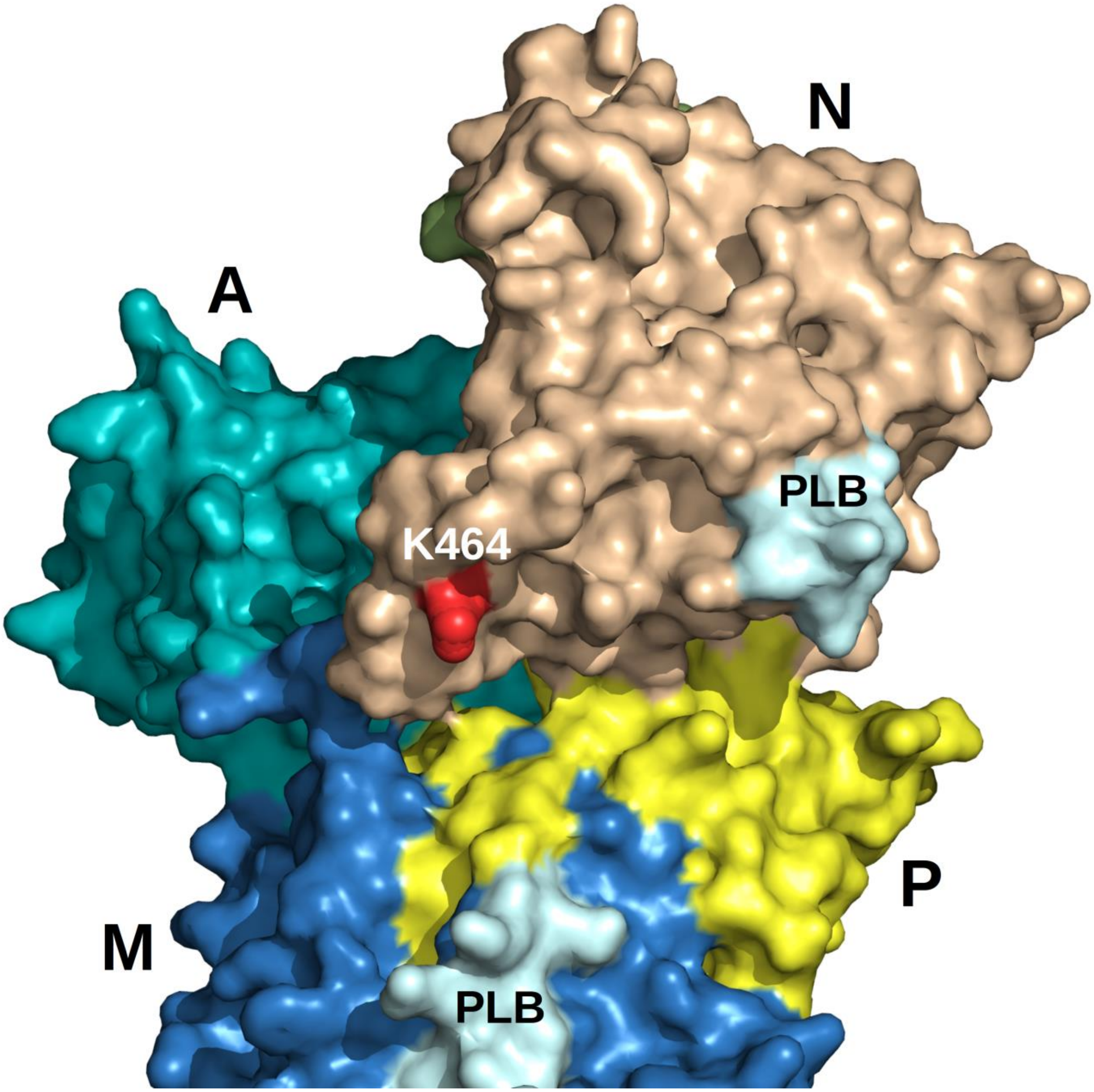

Figure S4. Surface neighborhood of K464. Domains N, P, A and M are represented in different colors, the putative phospholamban binding site (PLB) is in light blue. Residue K464 locates on the side of a cleft connecting domains N, P and M near the phospholamban binding site (PLB). Structure model generated with MODELLER based on template PDB ID: 3B9B chain A representing E2P conformational state.
